# A method for predicting target drug efficiency in cancer based on the analysis of signaling pathway activation

**DOI:** 10.18632/oncotarget.5119

**Published:** 2015-08-07

**Authors:** Artem Artemov, Alexander Aliper, Michael Korzinkin, Ksenia Lezhnina, Leslie Jellen, Nikolay Zhukov, Sergey Roumiantsev, Nurshat Gaifullin, Alex Zhavoronkov, Nicolas Borisov, Anton Buzdin

**Affiliations:** ^1^ Pathway Pharmaceuticals, Wan Chai, Hong Kong, Hong Kong SAR; ^2^ D. Rogachyov Federal Research Center of Pediatric Hematology, Oncology and Immunology, Moscow, Russia; ^3^ First Oncology Research and Advisory Center, Moscow, Russia; ^4^ Department of Genetics, Genomics, and Informatics, University of Tennessee Health Science Center, Memphis, TN, USA; ^5^ Pirogov Russian National Research Medical University, Department of Oncology, Hematology and Radiotherapy, Moscow, Russia; ^6^ Moscow State University, Faculty of Fundamental Medicine, Moscow, Russia; ^7^ Insilico Medicine, Inc., ETC, Johns Hopkins University, Baltimore, MD, USA; ^8^ Group for Genomic Regulation of Cell Signaling Systems, Shemyakin-Ovchinnikov Institute of Bioorganic Chemistry, Moscow, Russia

**Keywords:** cancer, response to target drug therapy, bioinformatic modeling, intracellular signaling pathway, personalized medicine

## Abstract

A new generation of anticancer therapeutics called target drugs has quickly developed in the 21^st^ century. These drugs are tailored to inhibit cancer cell growth, proliferation, and viability by specific interactions with one or a few target proteins. However, despite formally known molecular targets for every “target” drug, patient response to treatment remains largely individual and unpredictable. Choosing the most effective personalized treatment remains a major challenge in oncology and is still largely trial and error. Here we present a novel approach for predicting target drug efficacy based on the gene expression signature of the individual tumor sample(s). The enclosed bioinformatic algorithm detects activation of intracellular regulatory pathways in the tumor in comparison to the corresponding normal tissues. According to the nature of the molecular targets of a drug, it predicts whether the drug can prevent cancer growth and survival in each individual case by blocking the abnormally activated tumor-promoting pathways or by reinforcing internal tumor suppressor cascades. To validate the method, we compared the distribution of predicted drug efficacy scores for five drugs (Sorafenib, Bevacizumab, Cetuximab, Sorafenib, Imatinib, Sunitinib) and seven cancer types (Clear Cell Renal Cell Carcinoma, Colon cancer, Lung adenocarcinoma, non-Hodgkin Lymphoma, Thyroid cancer and Sarcoma) with the available clinical trials data for the respective cancer types and drugs. The percent of responders to a drug treatment correlated significantly (Pearson's correlation 0.77 *p* = 0.023) with the percent of tumors showing high drug scores calculated with the current algorithm.

## INTRODUCTION

For over six decades, chemotherapy has been a key treatment for many types of cancer, often with high rates of success. For example, the use of cisplatin-containing regiments in the treatment of testicular cancer turned ∼100% mortality to ∼90-95% disease-specific survival observed nowadays [1, 2]. However, many individual cases and types of cancer remain incurable or even unresponsive using standard chemotherapy approaches. Moreover, chemotherapy generally causes severe side effects, which significantly decrease the quality of life of a patient [3, 4]. The chemical compounds included in standard chemotherapy cocktails have numerous molecular targets in cancerous and normal cells, which makes it difficult to simulate and predict the activity of drug to an individual patient based on the molecular data, and in standard practice clinicians routinely use clinical or morphological predictive factors like stage, grade, proliferative activity, etc [5, 6]. These predictive factors are typically very inaccurate and not applicable for tracing the individual patient response to chemotherapy drugs and regimens.

To address specific activities of certain functionally relevant proteins and their aggregates frequently observed in cancer, a new generation of anticancer drugs was generated that target one or a few specific molecules in a cell [7]. This class of drugs consists mostly of specific monoclonal antibodies (Mabs) and low molecular weight kinase-inhibitor molecules (Nibs; [8]). At least fifty different anticancer target drugs have been approved by national food and drug administration (FDA) systems and present on the global pharmacological market today (e.g., accessible through Metadrug database, www.drugbank.ca).

The emergence of target drugs was beneficial for the treatment of several cancer types. For example, trastuzumab (anti-HER2 monoclonal antibody) and several other new anti-HER2 medications at least doubled median survival time in patients with metastatic HER2-positive breast cancer and improved 5-year survival in early stage disease to ∼90-95% [9, 10]. Interestingly, before the introduction of trastuzumab, HER2-positive cancers had the worst prognoses across all breast cancer subtypes, whereas now the situation is reverted [11]. Patients with melanoma (deadly skin cancer type) for decades had no treatment opportunities except dacarbazine chemotherapy, which resulted in <10% chance of very short-lasting (∼5-6 month) response and median survival less than a year. Now, in the case of BRAF-mutated tumor, they can receive vemurafenib (anti-BRAF target drug) and have ∼50% chance of response [12], or, irrespectively of BRAF mutation, ipilimumab (immune checkpoint inhibitor) with ∼20% chance of long-term (>5 years) disease control [13].

Importantly, the results of clinical trials clearly suggest that for many drugs considered inefficient for treatment of a given cancer type, a tiny fraction of the patients exists to whom these drugs can be of a significant benefit. For example, no benefit was seen in large randomized studies in cohort of unselected patients with non-small cell lung cancer after introduction of anti-EGFR drugs (gefitinib and erlotinib). But it was observed that ∼10-15% of the patients who participated in these studies survived unpredictably long. Further investigation revealed that all these patients had activating mutation of EGFR and that this mutation may predict response to the EGFR-targeting drugs. Indeed, contemporary studies showed tha t patients with EGFR-mutated tumors have the strongest advantage with these types of target therapy [14]. In the case of colorectal cancer, discovery of the role of KRAS mutation in the resistance to the EGFR-targeting antibody (cetuximab or panitumumab) helped to identify a group of patients that can benefit from this kind of treatment (patients with wild-type KRAS). Moreover, further studies demonstrated that for KRAS-mutated tumors (∼40% of colorectal cancer), anti-EGFR antibodies cause harm and decrease survival [15].

It is of great importance, therefore, to identify accurate predictive markers of target drug efficacy. Several clinical tests have been used to identify optimal personalized cancer treatments [16, 17]. These tests mostly utilize data on the expression of certain individual genes and on somatic mutations within these genes, as mentioned above. Alternatively, drugs can target abnormal fusion proteins frequently formed in cancer cells, such as chimeric fusion *BCR-ABL* and the respective drug imatinib [18, 19]. However, most of these predictor features profile only several biomarkers, cover only a minor fraction of target drugs, and are limited to a particular type of cancer. Somewhat more universal methods are required to rank the maximum number of existing drugs.

We propose that a shift in focus to the activation of intracellular signaling pathways in cancer may advance the development of such approach. We report here a method for predicting target drug efficacy based on a patient's cancer-specific patterns of signaling pathway activation (SPA), particularly for pathways including molecular targets of respective drugs. The enclosed algorithm operates with the so-called Pathway Activation Strength (PAS) value, which is a qualitative characteristic of pathway activity in a cancer sample. Several approaches were published previously by us and others to measure PAS based on large scale gene expression data; these may be used with either transcriptomes or proteomes. Khatri et al [20] classified those methods into three major groups: Over-Representation Analysis (ORA), Functional Class Scoring (FCS) and Pathway Topology (PT)-based approaches. ORA-based methods calculate if the pathway is significantly enriched with differentially expressed genes [21–23]. These methods have many limitations, as they ignore all non-differentially expressed genes and do not take into account many gene-specific characteristics. FCS-based approaches partially tackle aforementioned limitations by calculating fold change-based scores for each gene and then combining them into a single pathway enrichment score [24–26]. PT-based analysis also takes into account topological characteristics of each given pathway, assigning additional weights to the genes (for a review, see [27]). Recently, to account for gene expression variability within a pathway, another set of differential variability methods has been developed [28]. Differential variability analysis determines a group of genes with a significant change in variance of gene expression between case and control groups [29]. This approach was further extended and applied on the pathway level [28, 30, 31].

Recently, we developed OncoFinder, a new biomathematical method for pathway analysis [32] [33]. This method performs quantitative and qualitative analysis of signaling pathway activation. For each investigated sample, it performs a case-control pairwise comparison and calculates PAS, a value which serves as a qualitative measure of pathway activation. Unlike most other methods, this approach takes into account functional roles of all molecular participants of a pathway, and determines if the signaling pathway is significantly up- or down-regulated compared to the reference. Negative and positive overall PAS values correspond, respectively, to the inhibited or activated state of a pathway. OncoFinder is also, to our knowledge, a unique PAS calculating method, which provides output data with significantly reduced noise introduced by the experimental transcriptome profiling systems [33]. This feature enables characterization of the functional states of the transcriptomes and interactomes more accurately than prior methods. It was also shown to be efficient in finding new cancer biomarkers more stable than individual gene expression patterns [34]. To date, OncoFinder has demonstrated usefulness in several applications including leukemia and solid cancers [34–37], Hutchinson Gilford Disease [38] and Age-Related Macular Degeneration Disease [39].

Here, we present a novel approach for choosing an optimal personalized treatment for cancer patients based on high-throughput gene expression profiling of tumor samples. We introduce a Drug Score (DS) as a measure of effectiveness of a drug in a patient based on the rationale that a drug needs to compensate the changes in pathway activation/deactivation associated with cancer progression. We use clinical trials data to validate this scoring system.

We compared the distribution of the predicted drug efficacy scores for five drugs (Sorafenib, Bevacizumab, Cetuximab, Sorafenib, Imatinib, Sunitinib) and seven cancer types (Clear Cell Renal Cell Carcinoma, Colon cancer, Lung adenocarcinoma, non-Hodgkin Lymphoma, Lung Adenocarcinoma, Thyroid cancer and Sarcoma) with the available clinical trials data for the respective drugs and cancer types. The proportion of tumors for which high drug scores were calculated with the proposed algorithm correlated significantly with the percent of responders to a drug treatment (Pearson's correlation 0.77, *p* = 0.023).

## RESULTS

### Drug scoring algorithm

OncoFinder algorithm is based on the processing of Pathway Activation Strength (PAS) signatures of the cancer tissues under investigation. According to OncoFinder method, PAS is calculated using expression values of individual genes to investigate activation/deactivation of intracellular signaling pathways [33]. PAS is defined as a weighted sum of logarithmic case-to-normal ratios (*CNR*), i.e. fold-change of expression values of a gene in a biosample under study compared to average expression value in control samples. Two types of weighting coefficients are defined as indicators showing (*i*) if a protein CNR value exceeds the confidence interval (*BTIF*_n_, *beyond tolerance interval flag*); (*ii*) if a protein *n* represses (-1 value) or promotes (+1 value) signaling in the pathway *p* (*ARR*_np_, *activator/repressor role*); (*iii*) if a protein *n* is involved in pathway *p* (*NII*_np_, *node involvement index*).

Overall, PAS, or Pathway Activation Strength is calculated according to the following formula [33], where *p* represents the index of a pathway and *n* stays for the index of a protein:

$${\text{PAS}}_{p}\;=\;\underset{n}{\sum }{\text{NII}}_{\text{np}}{\text{ARR}}_{\text{np}}{\text{BTIF}}_{n}\text{lg}({\text{CNR}}_{n})$$

To construct a scoring function for a drug in a patient, or DS, we define the following indicators:

*AMCF* flag (*activation-to-mitosis conversion factor*) shows if the pathway activation promotes or inhibits mitosis and cell survival:

$${\text{AMCF}}_{p}\;=\{\begin{array}{l} 1,\text{pathway p promotes mitosis}\\ -1,\text{pathway p inhibits mitosis} \end{array}$$

*DTI* (drug-target index):

$${\text{DTI}}_{\text{dt}}\;=\;I(\text{drug d affects target protein t})\;=\;\{\begin{array}{l} 0,\text{ drug d does NOT affect target t}\\ 1,\text{ drug d affects target t} \end{array}$$

*NII* (node involvement index):

$${\text{NII}}_{\text{tp}}\;=\;I(\text{protein t is involved in pathway p})\;=\;\{\begin{array}{l} 0,\text{ protein t is NOT involved in pathway p}\\ 1,\text{ protein t is involved in pathway p} \end{array}$$

DS, which estimates the ability of a drug *d* to turn cancer-related pathological changes in the transcriptome of a tumor back to normal state is defined as follows:

$${\text{DS}}_{d}\;=\;\sum_{t}{\text{DTI}}_{\text{dt}}\sum_{p}{\mathrm{NII}}_{\text{tp}}{\text{AMCF}}_{p}\;{\text{PAS}}_{p}$$

In other words,

$${\text{DS}}_{d}\;=\;\sum_{t}I(\text{drug d affects protein t})\sum_{p}I(\text{protein t is involved in the pathway p}){\text{ AMCF}}_{p}{\text{PAS}}_{p}$$

Briefly, DS can be understood as a sum of Pathway Activation Scores (PAS) for the pathways in which the targets of a drug are involved. The same PAS can be summed up several times if a drug targets multiple proteins involved in the pathway.

The given formula for DS is in principle applicable for all target drugs, including small molecule inhibitors (Nibs) and monoclonal antibodies (Mabs). With a little modification, it might be also applied for scoring monoclonal antibodies attached to cytotoxic agents, so-called Killer Mabs. In that case, a different definition of Pathway Activation Strength can be used:

$${\text{PAS}}_{p}^{\text{killermab}}\;=\;\sum_{n}{\text{NII}}_{\text{np}}{\text{BTIF}}_{n}\text{lg}({\text{CNR}}_{n})$$

PAS for killer Mabs is a reduced case of PAS where AMCF and ARR indicators are set to 1. This reflects the fact that despite the real biological role of a protein *n* in signaling, its overexpression will attract cytotoxic agents to tumor cells.

### Validation of the Drug Scoring algorithm based on tumor expression profiling and clinical trials data

We calculated DS for 113 anticancer target drugs extracted from the DrugBank database (http://www.drugbank.ca/) for different cohorts of patients with different cancer types. We investigated gene expression in a total of 371 samples of tumors and control sets of corresponding normal tissues for 7 cancer types: Clear Cell Renal Cell Carcinoma, Colon cancer, Lung adenocarcinoma, non-Hodgkin Lymphoma, Thyroid cancer and Sarcoma (Table 1). Table S1 summarizes the best scoring targeted drugs for every cancer type under study. Distributions of the DS are shown in Supplementary Figure 1. In general, we observed that cancer types for which target drug therapy is known to be efficient show significantly higher drug scores: Clear Cell Renal Cell Carcinomas and Thyroid tumors demonstrated high scores for top-scoring drugs, whereas non-Hodgkin lymphomas and lung adenocarcinomas showed lower scores (Supplementary Table 1, Supplementary Figure 1).

**Table 1:** GEO gene expression datasets used in the study If normal samples were taken from different GEO dataset, its accession is shown in “Normal” column

Name	GEO AC (tumor)	GEO AC (normal)	Subtype	Number of patients: all (tumor)	Tissue type (normal)	Platform
**(A). Comparison of drugs scores with clinical trials results**						
Thyroid cancer	GSE33630		papillary thyroid carcinoma	94 (49)	thyroid	GPL570
non-Hodgkin lymphoma (NHL)	GSE12453		Diffuse large B-cell lymphoma	50 (25)	non-neoplastic B lymphocytes	GPL570
Renal cancer	GSE36895		Clear cell renal cell carcinoma	52 (29)	normal kidney cortices	GPL570
Lung cancer	GSE43580	GSE37768	adenocarcinoma (AC)	97 (77)	Peripheral lung tissue (non-smokers)	GPL570
Colon cancer	GSE23878		-	59 (35)	non-cancerous colorectal tissue	GPL570
Sarcoma	GSE31715	GSE28511	-	19 (16)	normal skeletal muscle tissue	GPL6947
**(B). Candidate drugs for Multiple Sclerosis**						
Multiple sclerosis	GSE21942			27 (12)	peripheral blood mononuclear cells	GPL570
**(C). Melanoma dataset with wt / V600E BRAF**						
Melanoma	GSE15605			74 (58): 31 wt + 20 V600E	Primary melanoma vs normal skin	GPL570

**Figure 1:**
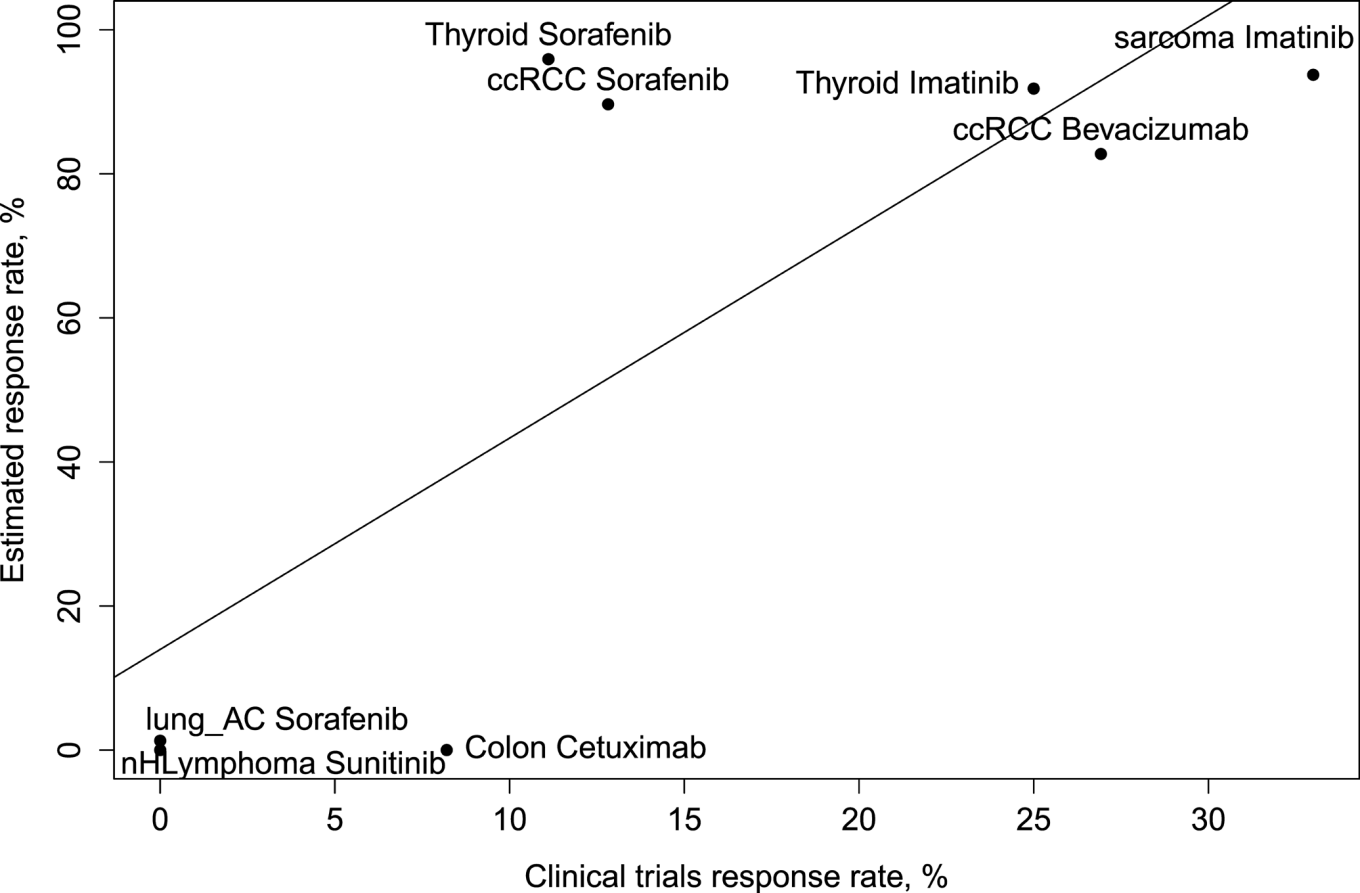
Scatter plot showing the percent of patients with a particular cancer type responding to a particular treatment (x-axis) in a clinical trial versus the percent of patients with a particular cancer type having the Drug Score for the particular drug above an arbitrary chosen cut-off value (250) (y-axis) ccRCC stands for Clear Cell Renal Cell Carcinoma, nHLymphoma for non-Hodgkin Lymphoma, lung AC for lung adenocarcinoma.

To investigate whether the DS successfully predicts treatment efficacy, we analyzed publically available clinical trials data from the ClinicalTrials database (clinicaltrials.gov) and different human cancer transcriptomes extracted from the Gene Expression Omnibus (GEO) database (http://www.ncbi.nlm.nih.gov/geo/). We checked if the number of patients responding and not responding to a treatment with a particular drug in a particular cancer type (Table 2) could be explained by the distribution of DS for that drug in patients with the particular cancer type. We assumed that the higher number of drug responders among the clinically investigated group of particular cancer patients should correspond to higher Drug Scores for the patients with same cancer type. Moreover, we assumed that a cut-off value could be chosen to distinguish the patients as responders or non-responders to a particular treatment according to their gene expression profile. We chose four cut-off values for DS between 100 and 500 to assess the correlation between the number of responders in a clinical trial and a predicted number of responders in a GEO dataset. To avoid multiple testing, only four cut-off values were tested (200, 250, 300, 350) and 250 was chosen as an optimal DS cut-off value providing the best correlation between fraction of responders in a clinical trial and fraction of patients with DS higher than the chosen cut-off. For the cut-off value of 250, we next calculated the percent of patients from a transcription profiling study showing higher DS than the cut-off rate. We observed that the fraction of patients with high DS correlated significantly with response rates in the respective clinical trials (Pearson's correlation 0.77, *p* = 0.023) (Figure 1).

**Table 2:** List of clinical trials analyzed in this study Patients showing complete or partial response were considered responders. ccRCC stands for Clear Cell Renal Cell Carcinoma, nHLymphoma for non-Hodgkin Lymphoma, lung AC for lung adenocarcinoma

Cancer type	Drug	% of responders	Clinical Study ID	Number of patients
ccRCC	Sorafenib	12.8	NCT00586105	39
ccRCC	Bevacizumab	26.9	NCT00719264	182
Colon	Cetuximab	8.2	NCT00083720	85
lung_AC	Sorafenib	0	NCT00064350	50
Thyroid	Imatinib	25	NCT00115739	8
Thyroid	Sorafenib	11.1	NCT00126568	18
nHLymphoma	Sunitinib	0	NCT00392496	15
sarcoma	Imatinib	33	NCT00090987	30

### Application of the drug scoring algorithm to multiple sclerosis datasets

To investigate whether the PAS-based DS can be efficiently used to rank drugs for diseases other than cancer, we tested this approach for Multiple Sclerosis (MS) patients. MS was chosen because anticancer target drugs, such as mitoxantrone, natalizumab or interferons, frequently show efficacy in treatment of this disease [40, 41]. MS is considered a systemic autoimmune disease, in which lymphocytes are immunoreactive against the patient's normal tissues [42]. We took the data on gene expression in peripheral blood mononuclear cells of MS patients and control patients (Table 1 section B). We hypothesized that the drugs which could compensate MS-specific changes in gene expression in peripheral blood mononuclear cells could be beneficial for patients suffering from MS. We prioritized the anticancer target drugs according to the mean DS they had in MS patients. The top five drugs identified in this assay are shown on Table 3. Even though the database contained only anti-cancer drugs, the three of five top drugs identified were previously studied as potential treatments of Multiple Sclerosis and showed considerable beneficial effects (Table 3).

**Table 3:** Drugs with the highest drug scores for MS patients

Drug	Mean Drug Score	Mentions of drug application for MS
Thalidomide	220.4	[43, 44]
Dasatinib	141.2	[45, 46]
Nilotinib	122.4	
Regorafenib	110.7	
Paclitaxel	103.7	[47]

### Drug score approach distinguishes between BRAF wild type and V600E mutants in melanomas

Unlike other approaches to ranking drugs for personalized cancer treatment, the algorithm suggested here does not require preliminary data on somatic mutations in tumors, thus substantially reduces the costs of analysis. While identifying the presence of mutations causing loss and gain of function of regulatory proteins is frequently an important step in predicting clinical outcome and treatment efficiency (e.g. *BRAF* V600E mutation) [12], we show here that a transcriptome-only approach also has the power to detect these changes at the gene expression level for downstream targets of the mutated regulator. Theoretically, the expression data may provide even more biologically meaningful results, as reliable methods for prediction of particular somatic mutations (e.g., gain-of-function) do not exist to date, and many mutations have limited or no phenotypic manifestations, depending heavily on the enclosing genomic context [48].

**Figure 2:**
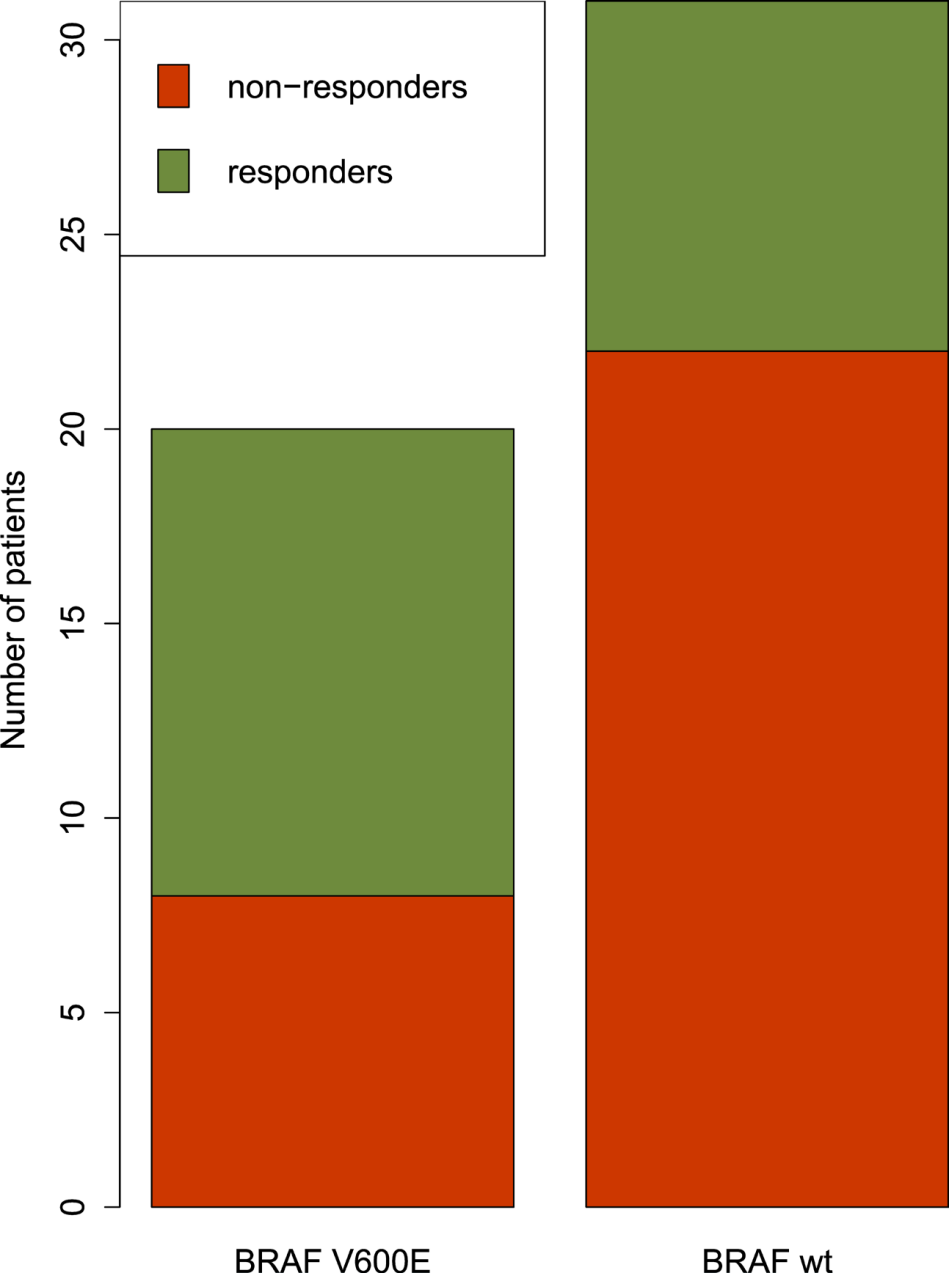
Cohort of tumors with *BRAF* V600E mutation (left bar) had significantly higher proportion of patients for whom Vemurafenib was predicted to be beneficial compared to a cohort with wild-type BRAF (right bar) Red bars show predicted non-responders and green bars show predicted responders (having non-zero DS for Vemurafenib).

To investigate the ability of our transcriptome-based drug scoring approach to distinguish between tumors harboring different driver mutations, we explored gene expression in melanoma patients. Vemurafenib is a target drug that is effective for melanoma tumors with V600E gain-of-function mutation in *BRAF* gene [12]. We compared DS for patients with wild type and V600E *BRAF* melanomas (Table 1 section C). We demonstrated that the percent of patients for whom Vemurafenib was expected to be beneficial (those having a positive DS for this drug) was significantly higher for the cohort of BRAF V600E-mutated tumors (p(Fisher) = 0.042, Figure 2).

The reason why an expression-based approach works well in this case is likely due to the ability to detect expression changes introduced by transcriptional reprogramming driven by the molecular consequences of V600E *BRAF* mutation. Of note, activation profiles of several molecular pathways correlated strongly with the mutation carrier status for the patients under investigation (Supplementary Table 2).

## DISCUSSION

Here we present a novel biomathematical method, which has a potential to be universal tool for predicting drug efficacy in the treatment of cancer via characterization of tumor-related patterns in intracellular signaling. It may have wide applicability, not only across the range of cancer types, but also to individual samples toward the goal of personalized cancer treatment. Unlike most part of other approaches to drug scoring in cancer, the current method does not require data on somatic mutations in tumors, thus substantially reducing the costs of an assay. Rather, it relies on advanced gene expression analysis. Although the presence of mutations causing loss and gain of function of certain regulator proteins is an important factor in the prediction of clinical outcome and treatment efficacy, a transcriptome-only approach will still potentially detect these changes as expression changes in downstream targets of the mutated regulator. Moreover, because reliable methods for predicting the effects of many specific somatic mutations (e.g. gain of function) do not yet exist, results based on expression data may be more biologically meaningful. As a proof of concept, we have demonstrated that our approach predicts the efficacy of Vemurafenib in melanoma samples without knowing the mutation status of BRAF; indeed, the prediction corresponded to presence of V600E gain-of-function mutation. For several other cancer types, we demonstrated the statistically significant advantage of this approach in identification of the top target drugs efficient for the respective cancer patients. On the model of multiple sclerosis, we showed that the current method of drug scoring is applicable also to non-tumor diseases. The approach we report here is platform-independent, i.e. any kind of high-throughput proteomic and transcriptomic data may be used to estimate gene expression.

## MATERIALS AND METHODS

### GEO expression profiles of tumors

The following datasets were analyzed in the study: GSE26886, GSE33630, GSE12453, GSE12460, GSE46170, GSE50161, GSM904985, GSE43580, GSE43580, GSE23878, GSE16515, GSE31189 (Supplementary Table 1). All the data were obtained with Affymetrix Human Genome U133 Plus 2.0 Array (GEO platform GPL570). The datasets contained tumor samples and normal samples of corresponding tissues from the same or different individuals.

### Preprocessing of microarray data

Raw microarray data (CEL files) were preprocessed with in R (version 3.1.0) using GCRMA method from *affy* package [49].

### Clinical trials data

A complete list of clinical trials analyzed in this study can be found in Table 1.

### Databases of known targeted drugs and pathways

Source datasets. The signalling pathways knowledge base developed by SABiosciences (http://www.sabiosciences.com/pathwaycentral.php) was used to determine structures of intracellular pathways, which were used for OncoFinder as described previously [33].

### Calculation of PAS and DS

Drug Score and Pathway Activation Strength values were calculated as described in the Results section with the following parameters: for each sample, only gene expression values which (1) were significantly (p>0.05) different from the distribution of expression in the set of control samples and (2) had CNR cancer-to-normal ratio outside of the interval between 0.66 and 1,5, thus leaving only the genes significantly different in terms of expression from control samples both in terms of statistical significance and magnitude

## SUPPLEMENTARY MATERIAL FIGURES AND TABLES


