# Supplementary material for: A method for predicting target drug efficiency in cancer based on the analysis of signaling pathway activation
Source: Oncotarget. 2015 Aug 7;6(30):29347–56. doi: 10.18632/oncotarget.5119 (PMC4745731; doi:10.18632/oncotarget.5119)
Supplement: Supplementary file 1 [file oncotarget-06-29347-s001.pdf]

A method for predicting target drug efficiency in cancer based on the analysis of signaling pathway activation

Supplementary Material

Clear cell renal cell carcinoma

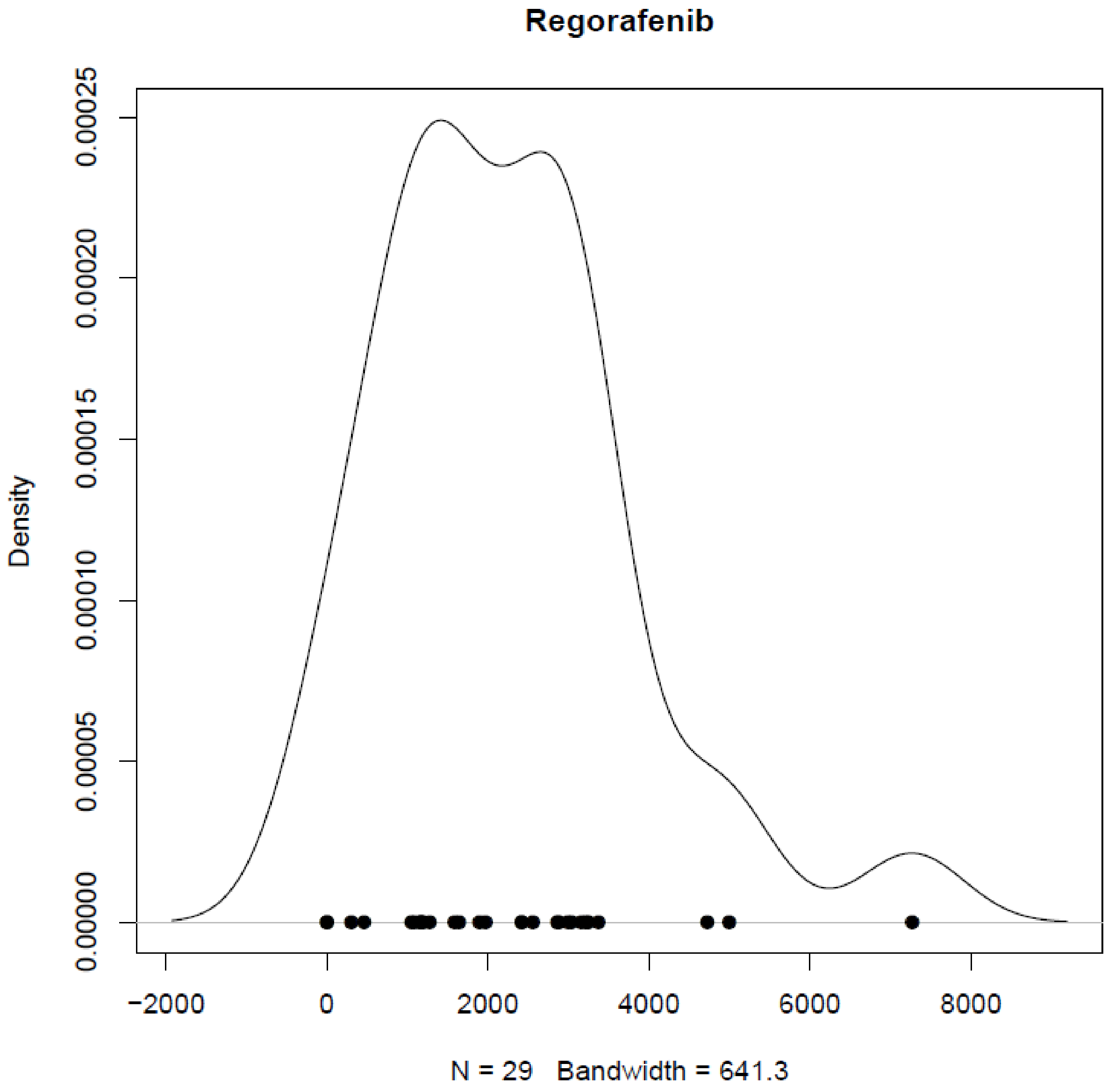

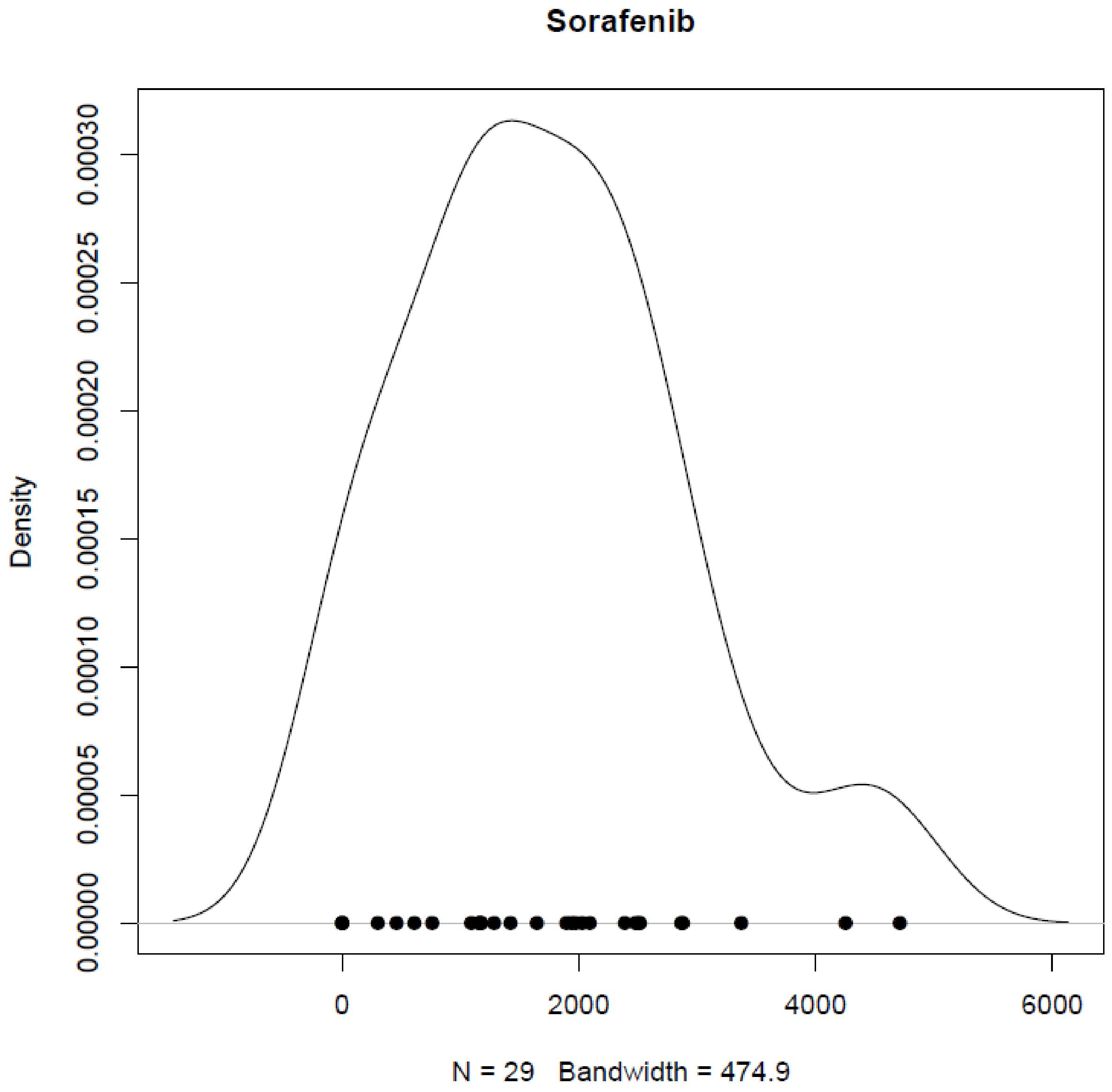

Thyroid cancer

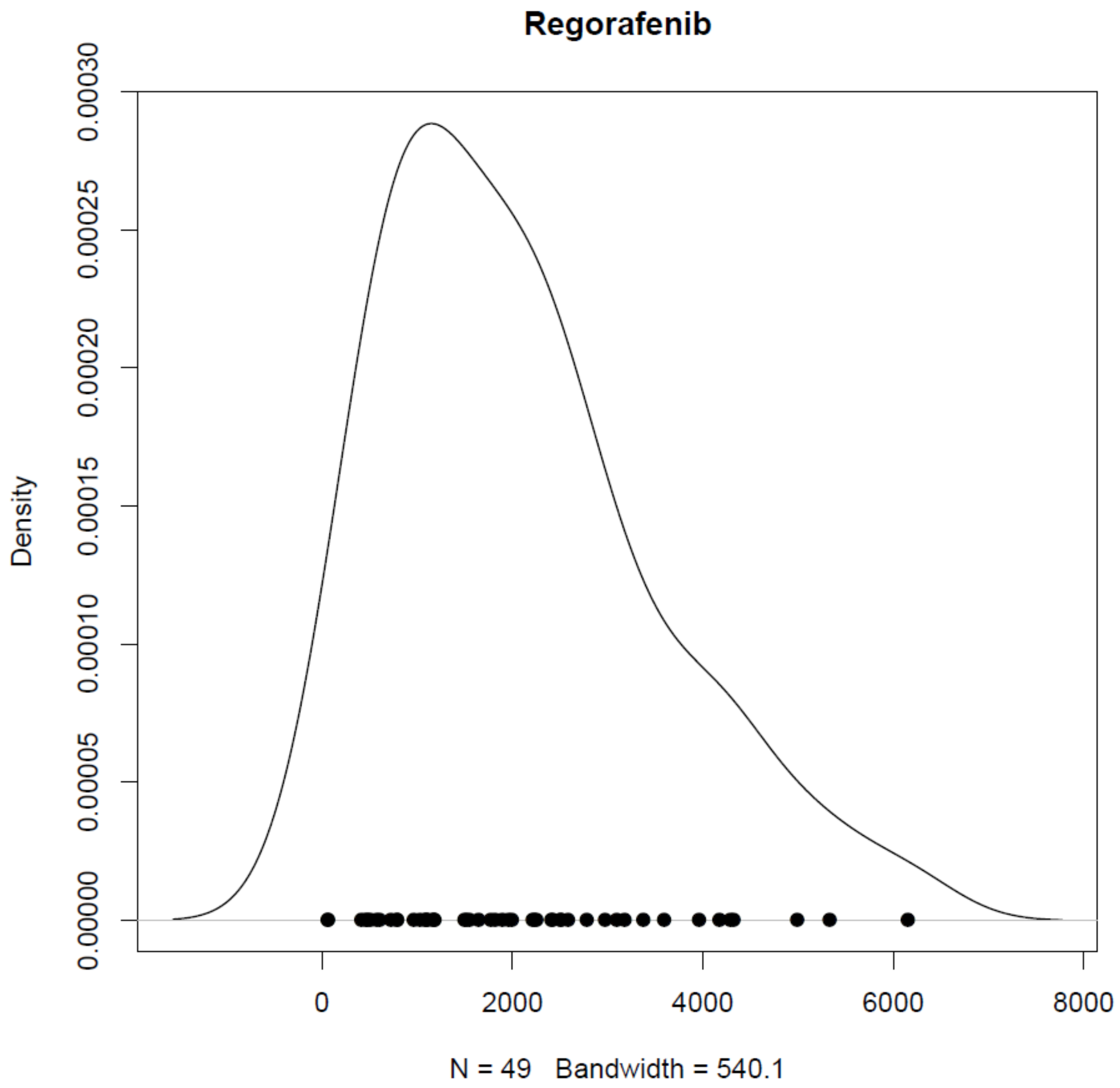

Regorafenib
Density
0.00000
0.00005
0.00010
0.00015
0.00020
0.00025
0.00030
0
2000
4000
6000
8000
N = 49   Bandwidth = 540.1

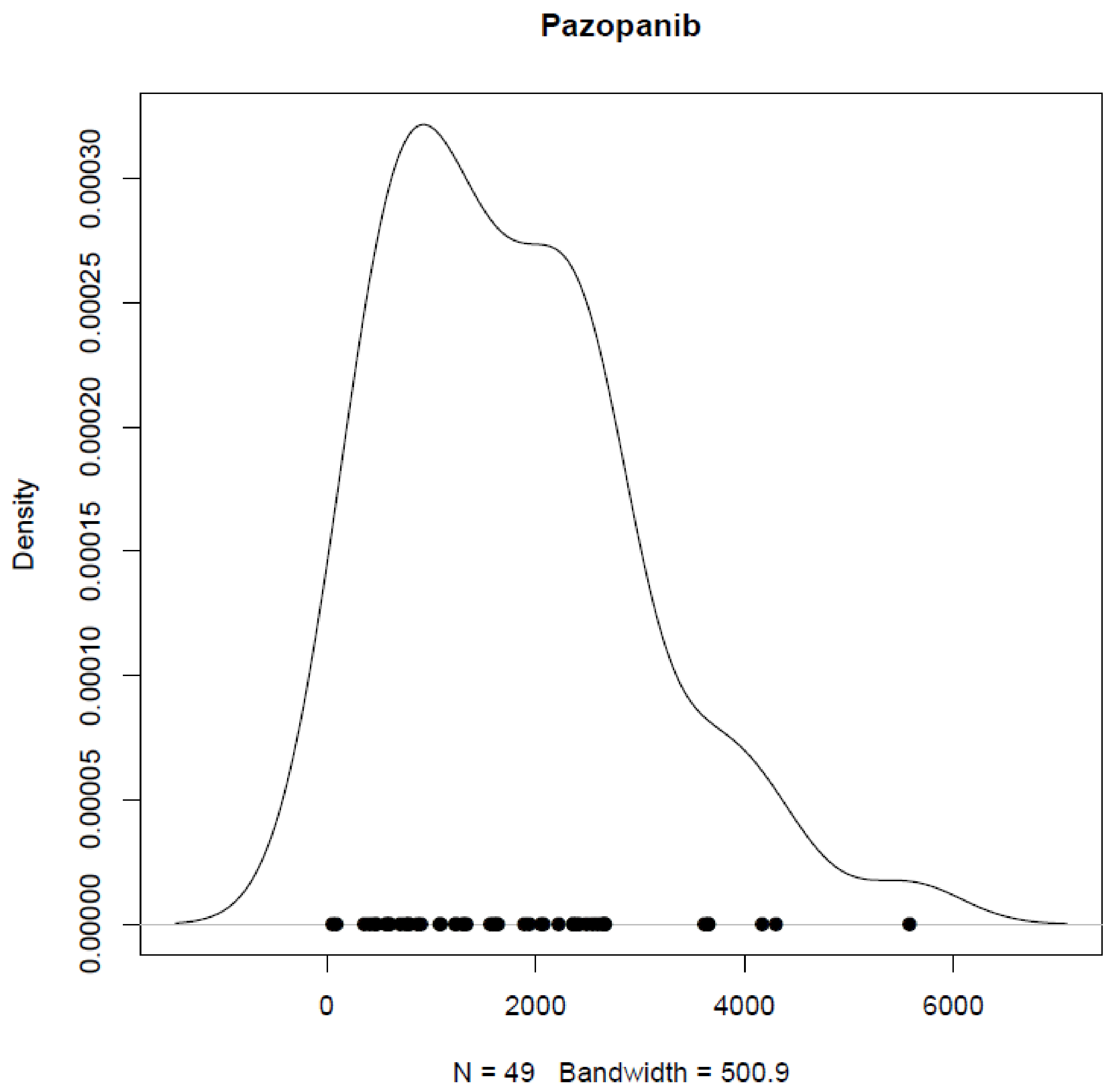

Pazopanib
Density
0.00000
0.00005
0.00010
0.00015
0.00020
0.00025
0.00030
0
2000
4000
6000
N = 49 Bandwidth = 500.9

Lung cancer

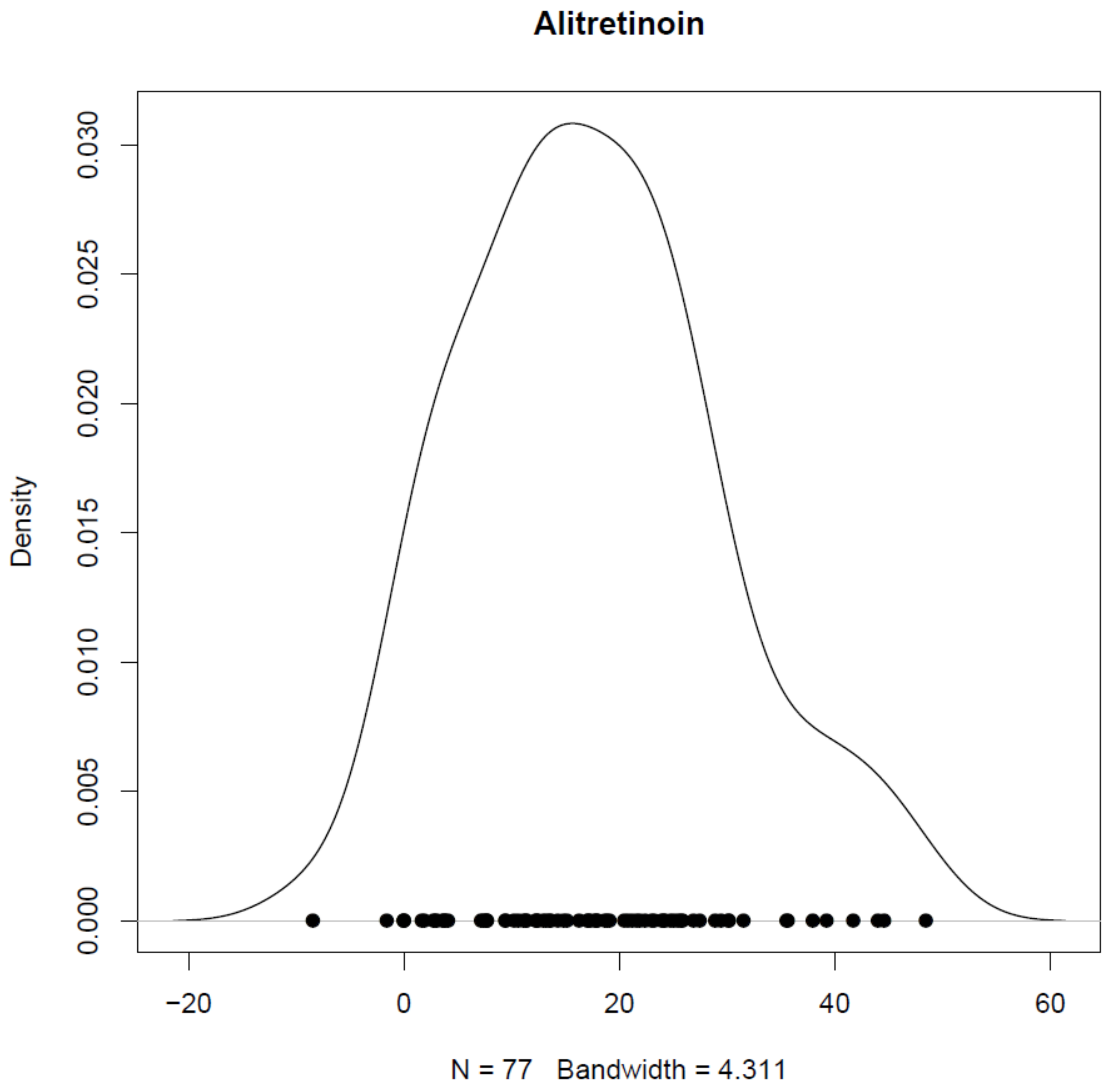
Alitretinoin
Density
0.000
0.005
0.010
0.015
0.020
0.025
0.030
−20
0
20
40
60
N = 77   Bandwidth = 4.311

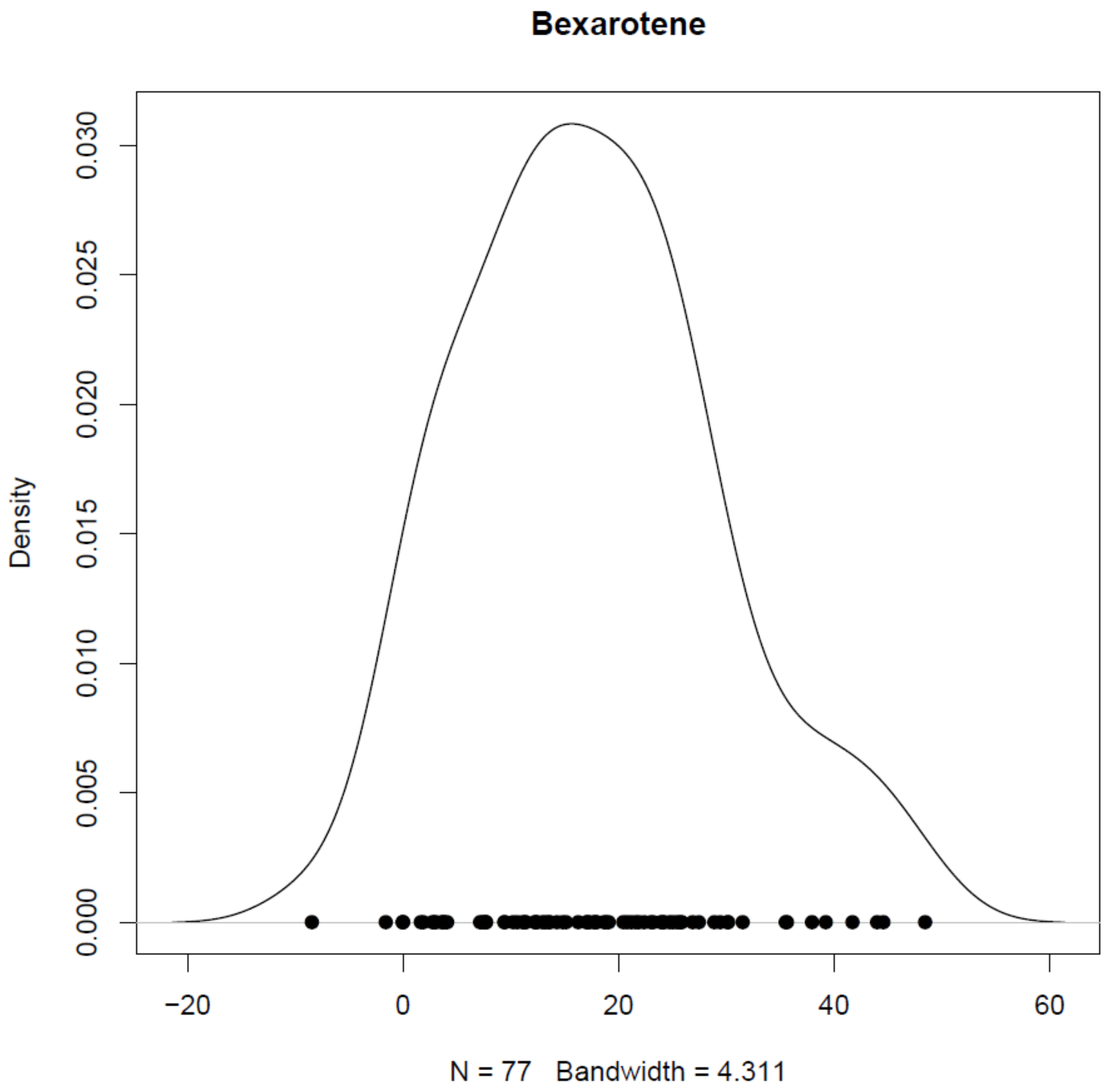

Non-Hodgkin lymphoma

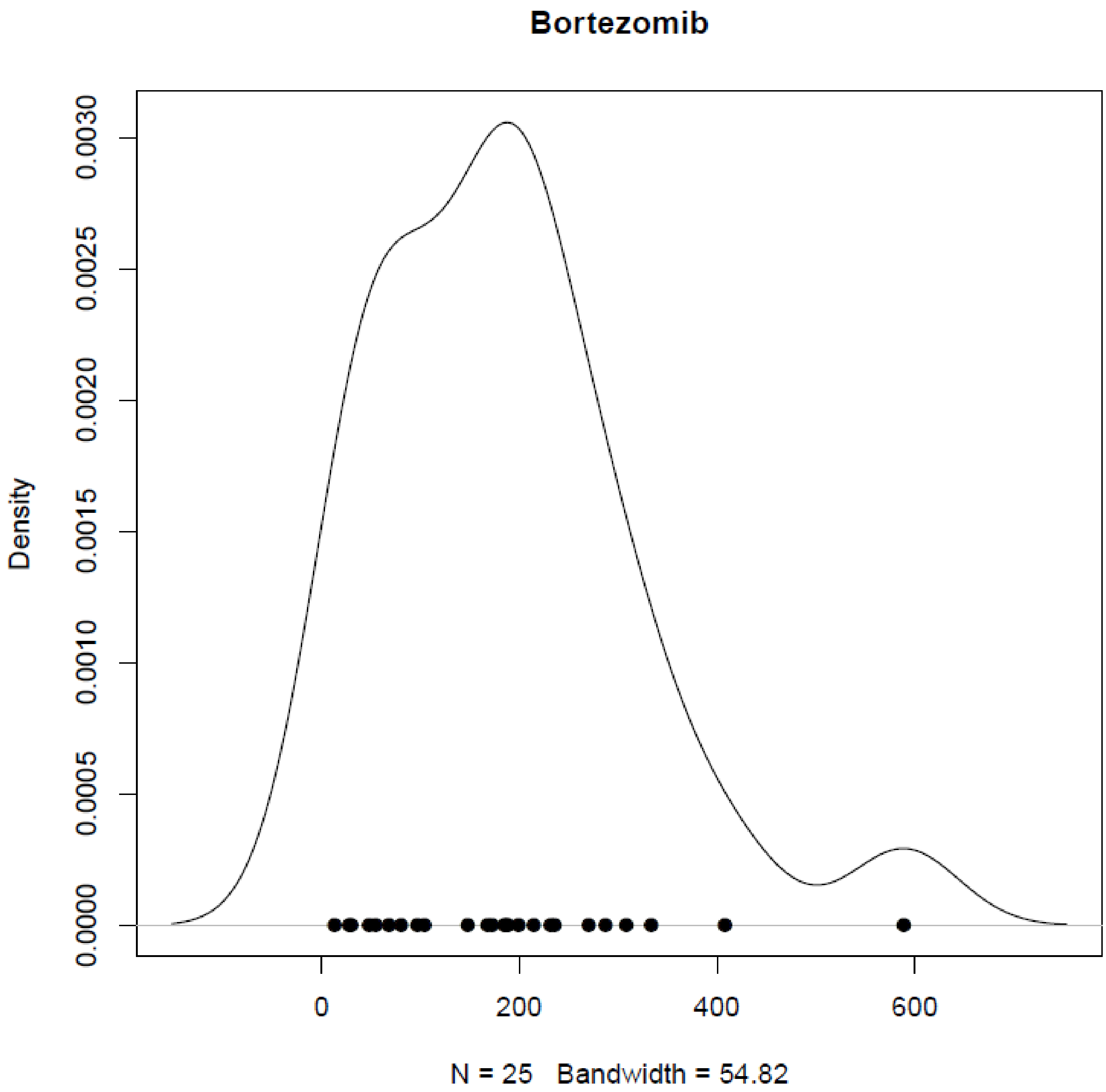

Bortezomib
Density
0.0000
0.0005
0.0010
0.0015
0.0020
0.0025
0.0030
0
200
400
600
N = 25 Bandwidth = 54.82

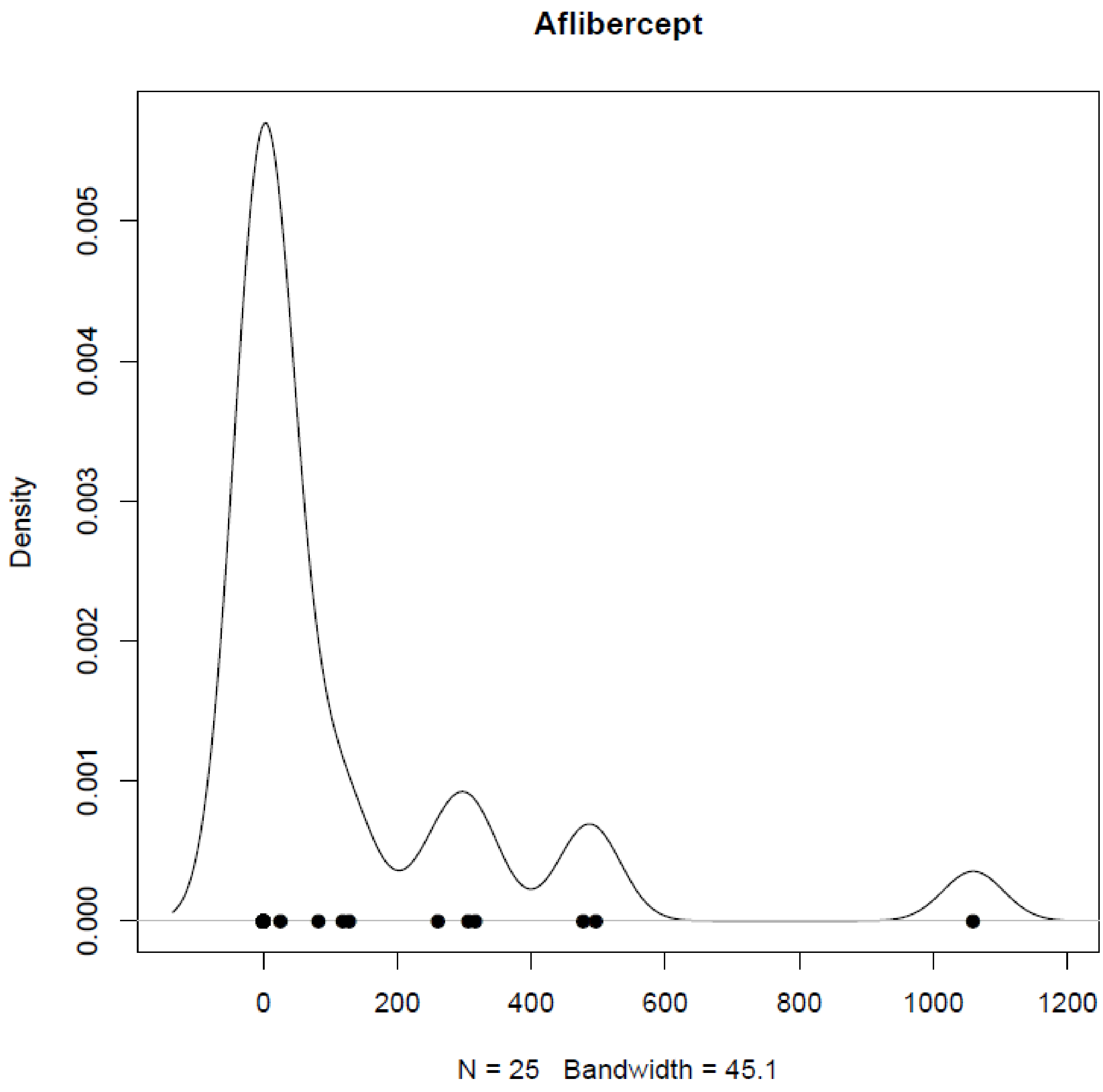

Colon cancer

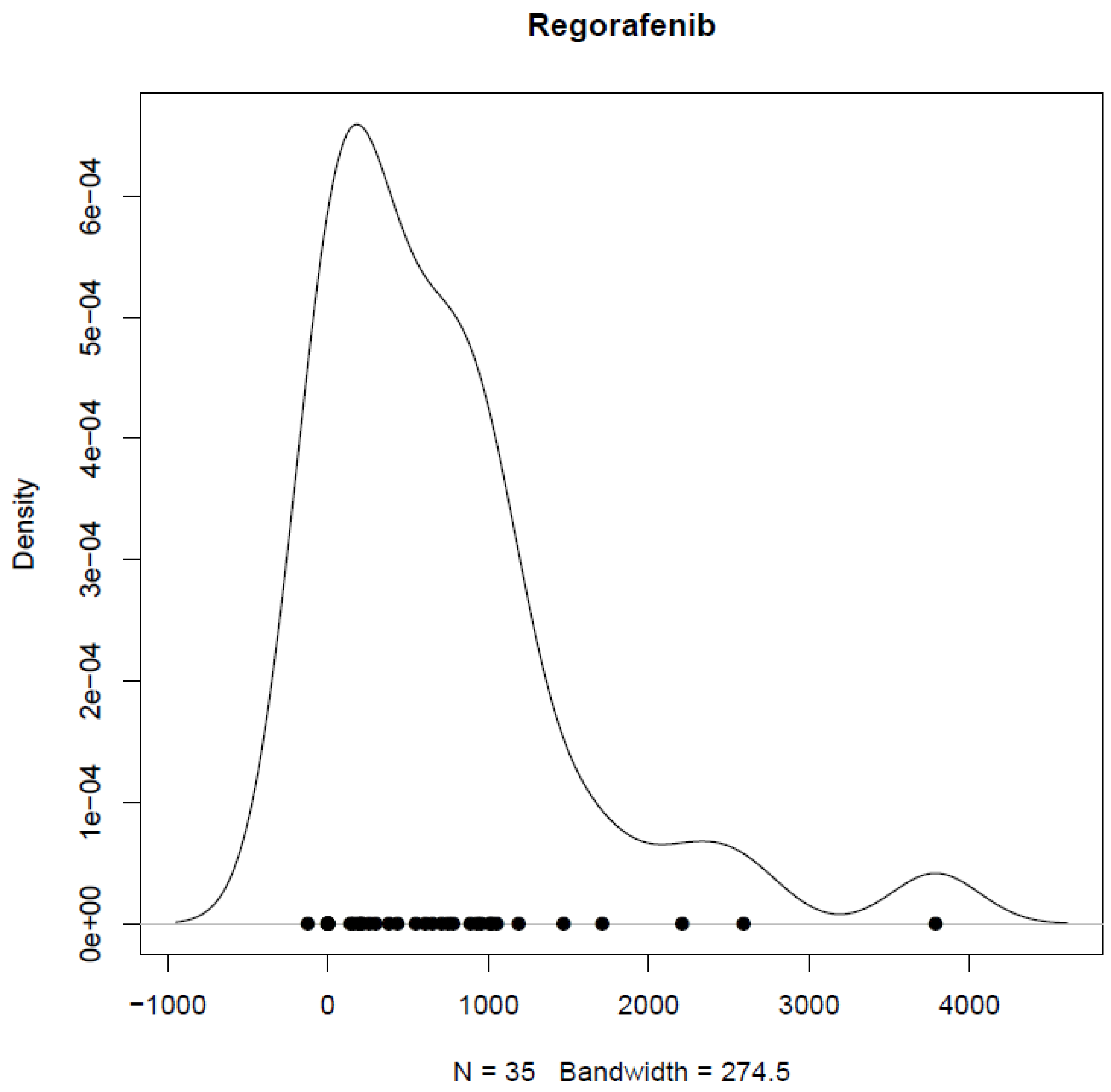

Regorafenib
Density
0e+00
1e−04
2e−04
3e−04
4e−04
5e−04
6e−04
−1000
0
1000
2000
3000
4000
N = 35   Bandwidth = 274.5

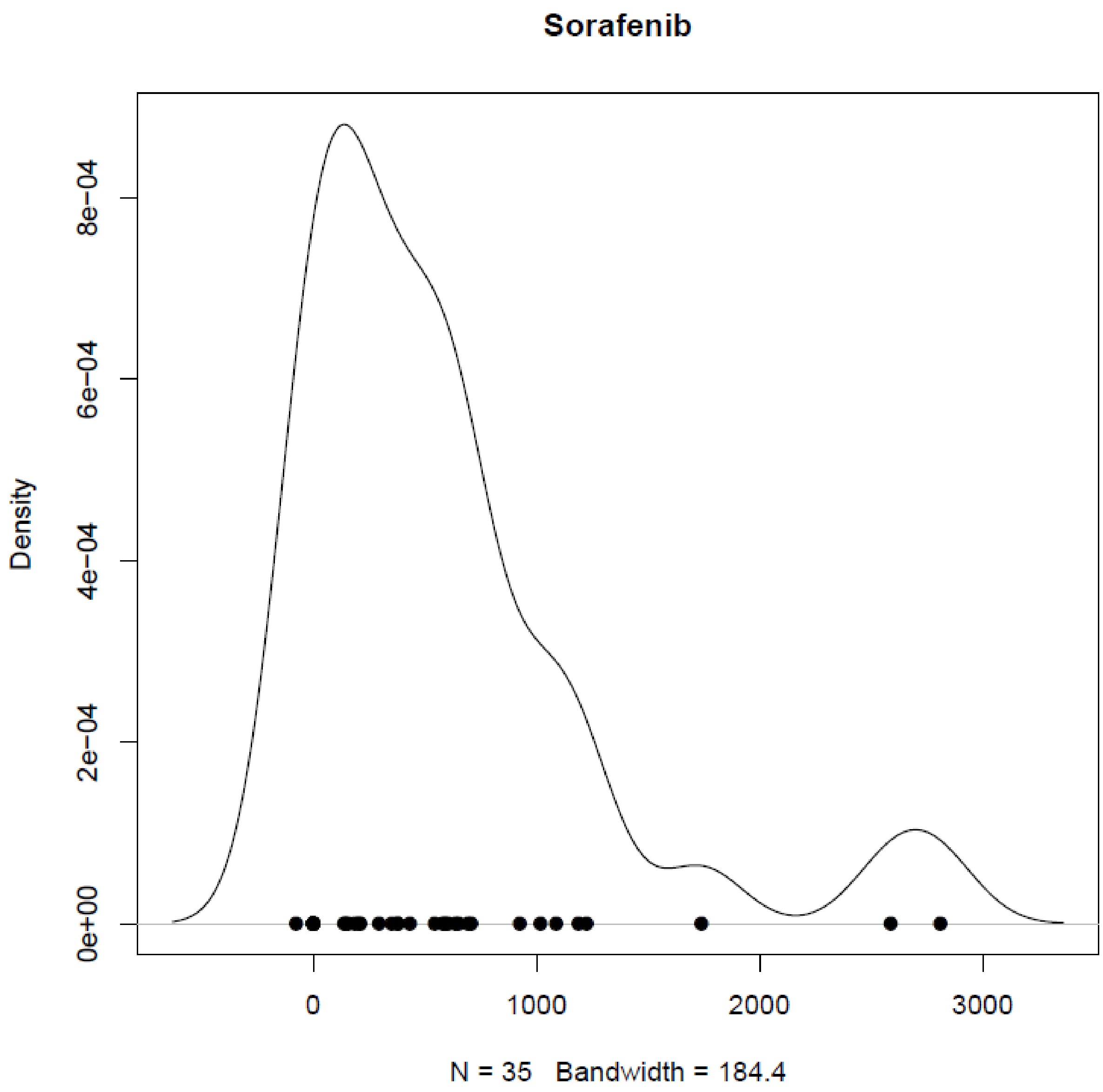
Sorafenib
Density
0e+00
2e−04
4e−04
6e−04
8e−04
0
1000
2000
3000
N = 35   Bandwidth = 184.4

Sarcoma

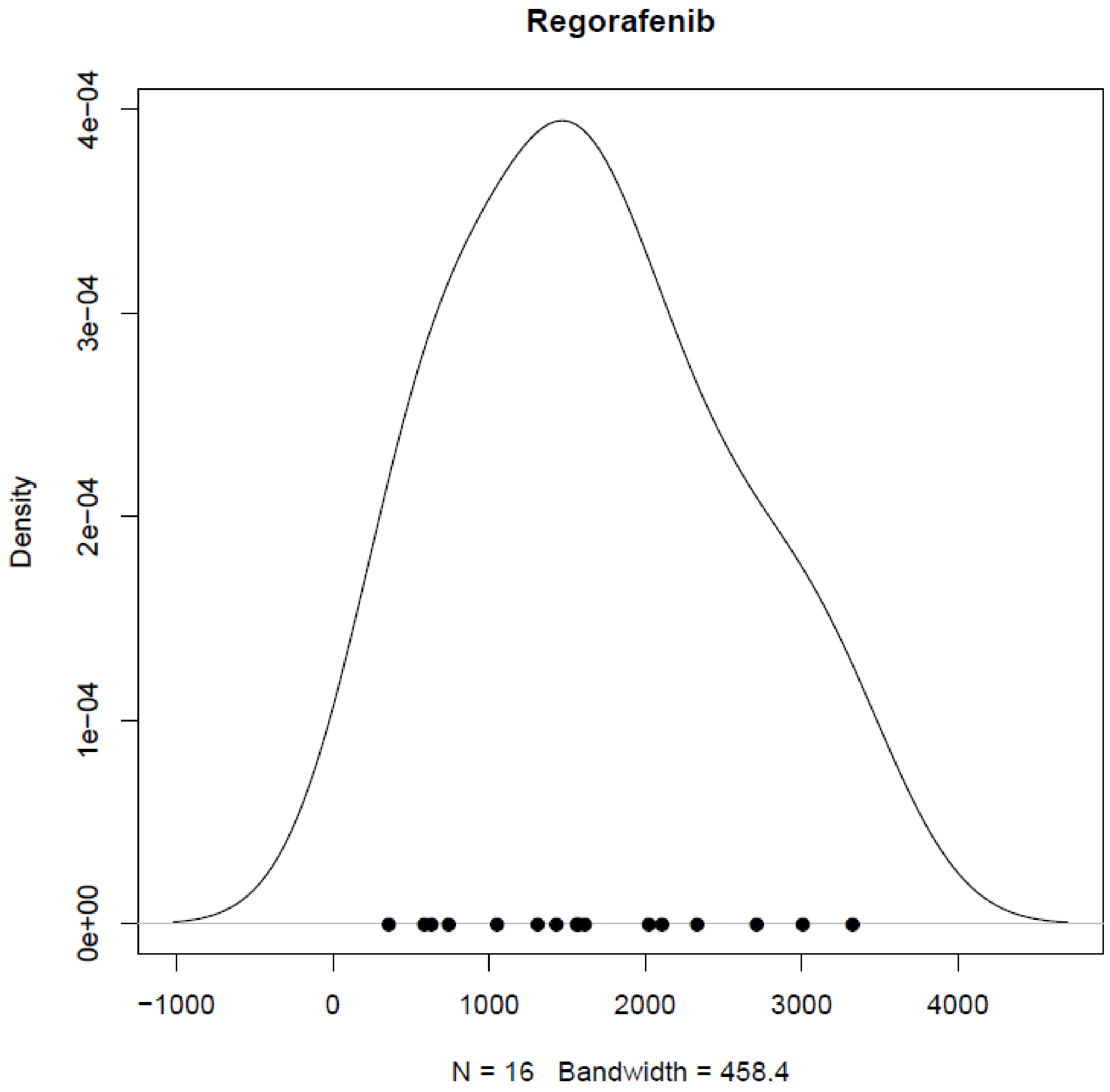

Regorafenib
Density
4e−04
3e−04
2e−04
1e−04
0e+00
−1000
0
1000
2000
3000
4000
N = 16 Bandwidth = 458.4

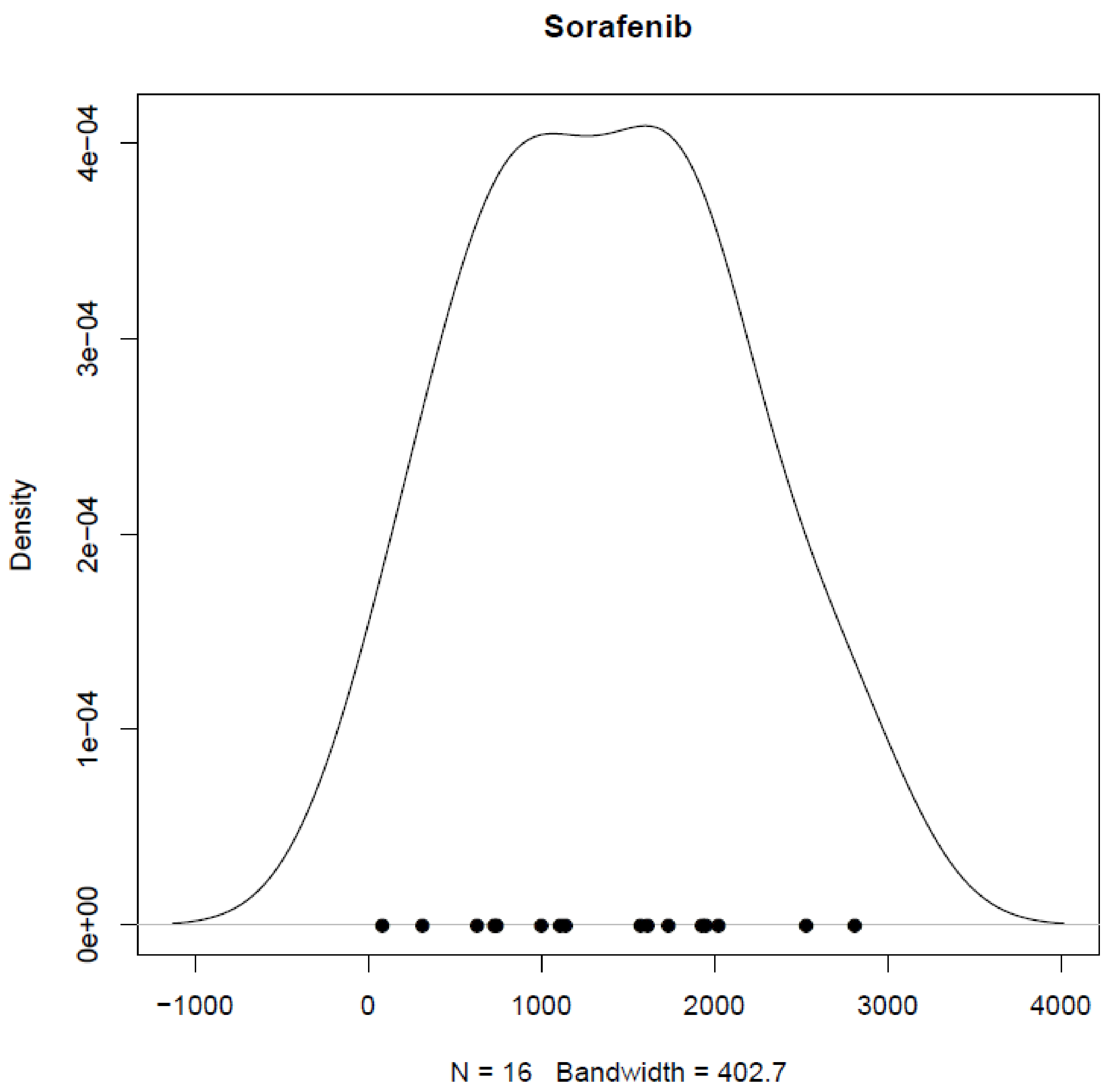

Figure S1. Distributions of Drug Scores for top drugs for every cancer type analyzed. X-axis corresponds to Drug Score, each point represents a tumour sample, density function summarizes the distribution of the pouints

Supplementary figures and tables

Table S1. Top drugs with their respective mean drug scores for each cancer type analyzed.

Clear cell renal cell carcinoma		Thyroid cancer	
**drug**	**meanScore**	**drug**	**meanScore**
Regorafenib	2244.436	Regorafenib	2065.974
Sorafenib	1727.931	Pazopanib	1775.138
Ziv-aflibercept	1593.795	Sorafenib	1746.129
Aflibercept	1578.758	Sunitinib	1443.551
Cabozantinib	1245.862	Imatinib	1129.987
Pazopanib	857.6003	Cabozantinib	1047.283
Sunitinib	851.7679	Dasatinib	1022.763
Crizotinib	788.191	Crizotinib	662.1324
Trametinib (Mekinst)	755.4259	Vandetanib	475.1567

non-Hodgkin lymphoma		Lung adenocarcinoma	
**drug**	**meanScore**	**drug**	**meanScore**
Bortezomib	186.02	Alitretinoin	17.47636
Aflibercept	130.7308	Bexarotene	17.47636
Ziv-aflibercept	130.7308	Tretinoin	17.47636
Regorafenib	123.1908	Fludarabine	16.66974
Sorafenib	113.4812	Alemtuzumab	9.759091
Dabrafenib	86.5168	Denileukin diftitox	4.057792
Thalidomide	58.372	Drostanolone	1.354026
Carfilzomib	53.1488	Methyltestosterone	1.354026
Trametinib (Mekinst)	48.5912	Ipilimumab	1.177532

Colon cancer		Sarcoma	
**drug**	**meanScore**	**drug**	**meanScore**
Regorafenib	713.6106	Regorafenib	1647.448
Sorafenib	583.384	Sorafenib	1369.189
Pazopanib	520.5677	Imatinib	979.8888
Sunitinib	520.5677	Pazopanib	965.6819
Bortezomib	441.4789	Sunitinib	965.1581
Dasatinib	439.6583	Ziv-aflibercept	810.2944
Imatinib	426.5074	Aflibercept	803.0869
Cabozantinib	316.144	Dasatinib	768.02
Ziv-aflibercept	289.9069	Dabrafenib	373.6506

Table S2. Top pathways associated with BRAF V600E mutations

Pathway	P-value (t-test, if PAS values differ between BRAF V600E and wt samples)	Difference between mean PAS (V600E-wt)
Integrin_Signaling_Pathway_Translocation_to_the_Nucleus	0.012	1.13
Caspase_Cascade_Apoptosis	0.015	-2.09
ATM_Pathway_MDMX_Ubiquitination_&_Degradation	0.019	0.61
